# The invasive blue crab *Callinectessapidus* Rathbun, 1896 (Decapoda, Portunidae) is rapidly expanding its distributional range in the north-western Mediterranean coast of Morocco

**DOI:** 10.3897/BDJ.12.e115875

**Published:** 2024-01-22

**Authors:** Fatima Zahra Hamiche, Mustapha Aksissou

**Affiliations:** 1 Laboratory Ecology, Systematics, Conservation of Biodiversity, LESCB, URL-CNRST N°18, Abdelmalek Essaadi University, Faculty of Sciences, Tetouan, Morocco Laboratory Ecology, Systematics, Conservation of Biodiversity, LESCB, URL-CNRST N°18, Abdelmalek Essaadi University, Faculty of Sciences Tetouan Morocco

**Keywords:** invasive species, Portunidae, bio-invasion, western Mediterranean Sea, Morocco

## Abstract

In this study, we report the first occurrence of *Callinectessapidus* in the rivers of ‘Oued Tani’ (Martil) and ‘Oued Negro’ (Fnideq), based on 127 individuals of the blue crab caught from November 2022 to August 2023. Additionally, we were able to determine the potential consequences of *C.sapidus* on the indigenous species as well as the socioeconomic implications on artisanal fisheries activities. This research highlights further data that reinforces recent findings on recorded blue crab from various locations along the Moroccan coastline.

## Introduction

Non-indigenous species (NIS, also known as non-native, alien or allochthonous) are a serious threat to coastal ecosystems including estuaries and lagoons as well as marine biodiversity (Saccà 2016). The blue crab *Callinectessapidus* Rathbun, 1896, (Crustacea, Decapoda, Brachyura, Portunidae) originates from the western Atlantic coasts. In fact, the native geographical distribution area of the Atlantic blue crab includes the Antilles, the Gulf of Mexico in addition to Bermuda and extends from Nova Scotia in Canada, Maine and northern Massachusetts to northern Argentina (Williams 1974, Nehring 2011, Castriota et al. 2012), although recent climate changes appear to favour *C.sapidus*' metabolic response and proliferation around the world (Marchessaux et al. 2022). In the Mediterranean Sea, *C.sapidus* is recognised as an Invasive Alien Species (Mancinelli et al. 2016) and categorised amongst the top 100 worst problematic Invasive Alien Species due to the large number of records (Streftaris and Zenetos 2006). The first recorded occurrence of *Callinectessapidus* from North Africa in Morocco was discovered near the Marchica Lagoon at Nador in 2017 (Chartosia et al. 2018). Recently, this species was reported in the ports of Tangier Med (Chairi and González-Ortegón 2022). Additionally, it has been observed in other nearby southern Mediterranean African countries including Algeria (Kara and Chaoui 2021) and Tunisia (Mili et al. 2020). According to Simberloff et al. (2013), Invasive Alien Species (IAS) represent a serious danger to marine biodiversity and coastal ecosystems because of their adverse interactions with other native species in areas that have been invaded (Cardeccia et al. 2018), additionally to further detrimental effects, particularly on artisanal fishing operations (Nehring 2011). Therefore, the present paper reports the appearance and the extent of expansion of *C.sapidus* along the north-western Mediterranean coast of Morocco and highlights the potential negative impacts on coastal ecosystems and biodiversity as well as on artisanal fishing activities.

## Materials and methods

**Figure 1.**
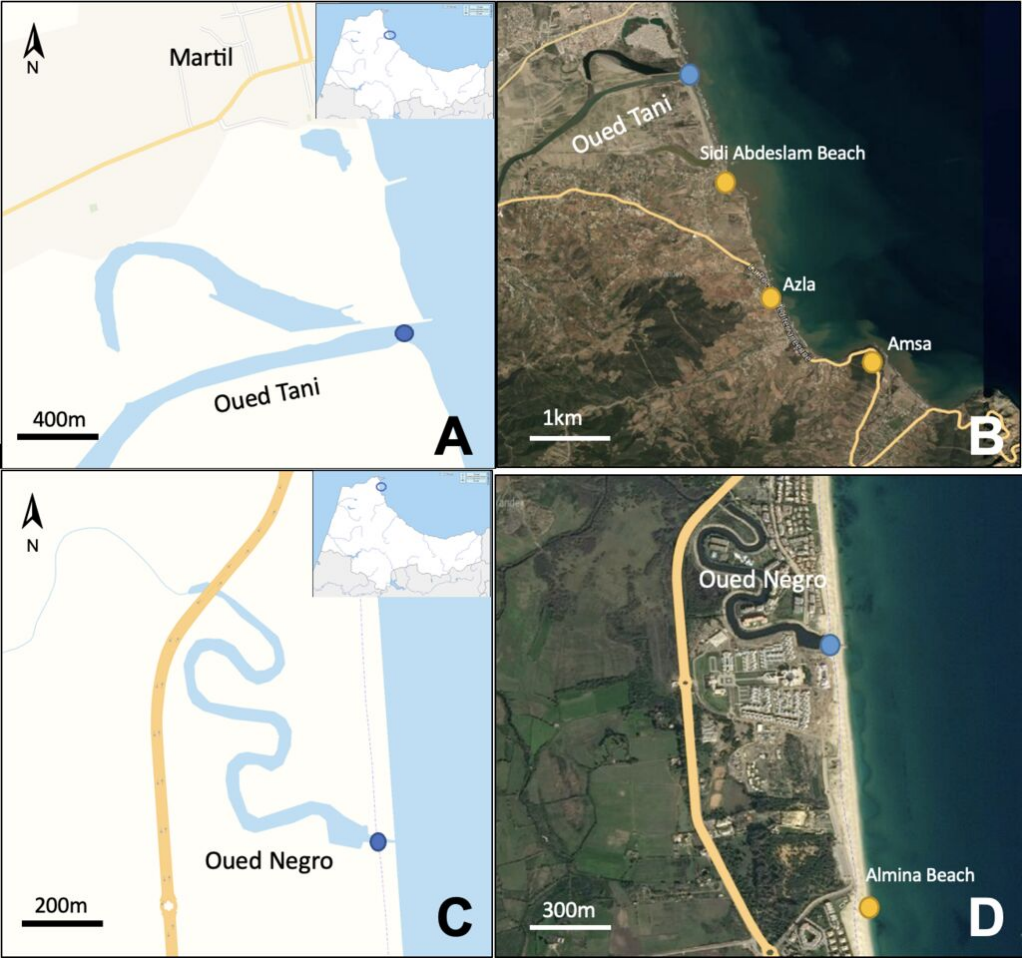
Map showing the localities of the study sites. **A** Mouth of Tani River (Martil); **B** Locations of the occurrence of *Callinectessapidus* in Amsa, Azla and Sidi Abdeslam Beaches (yellow dots) and Oued Tani Martil (blue dot); **C** Mouth of Negro River (Fnideq); **D** Locations of the occurrence of *Callinectessapidus* in Almina Beach (yellow dot) and Oued Negro Fnideq (blue dot).

The presence of the blue crab was reported, based on individuals caught with trammel nets and fishing rods by professional and recreational fishermen in shallow waters on muddy or muddy-sandy bottoms at depths ranging between 2 and 7 m. Specimens were captured from November 2022 to August 2023 in several localities distributed adjacent to the western Mediterranean coast of Morocco, including nearshore brackish waters and inland waters in order to track the expansion of this species. These locations included the following: Martil (Oued Tani), Fnideq (Oued Negro), Sidi Abdeslam, Azla, Amsa and Almina (Fig. 1). The individuals collected were directly transferred alive to the laboratory to be measured and classified by morphological characteristics, regarding their carapace length (CL), carapace width (CW) and weight (W), using appropriate identification guides by Williams (1974) and Millikin and Williams (1984). Additionally, through the clearly discernible pleon and telson anatomy depicting sexual dimorphism, caught individuals were sexed. The ovigerous status of the females was determined. Furthermore, the economic and social effects of *C.sapidus* on local fisheries was determined in Martil (Oued Tani) that was highly contaminated by blue crab (Suppl. material 1).

## Results

**Figure 2.**
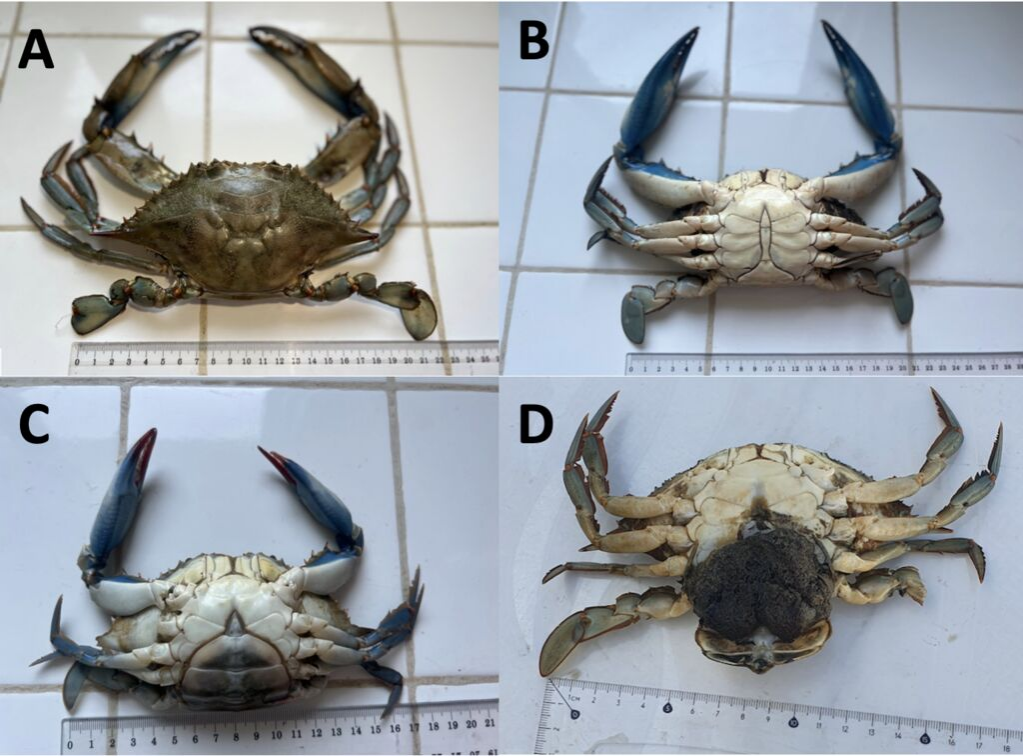
*Callinectessapidus* specimens caught in the western Mediterranean coast of Morocco. **A**; **B** dorsal and ventral views of male; **C** ventral view of female; **D** ventral view of ovigerous female.

**Figure 3.**
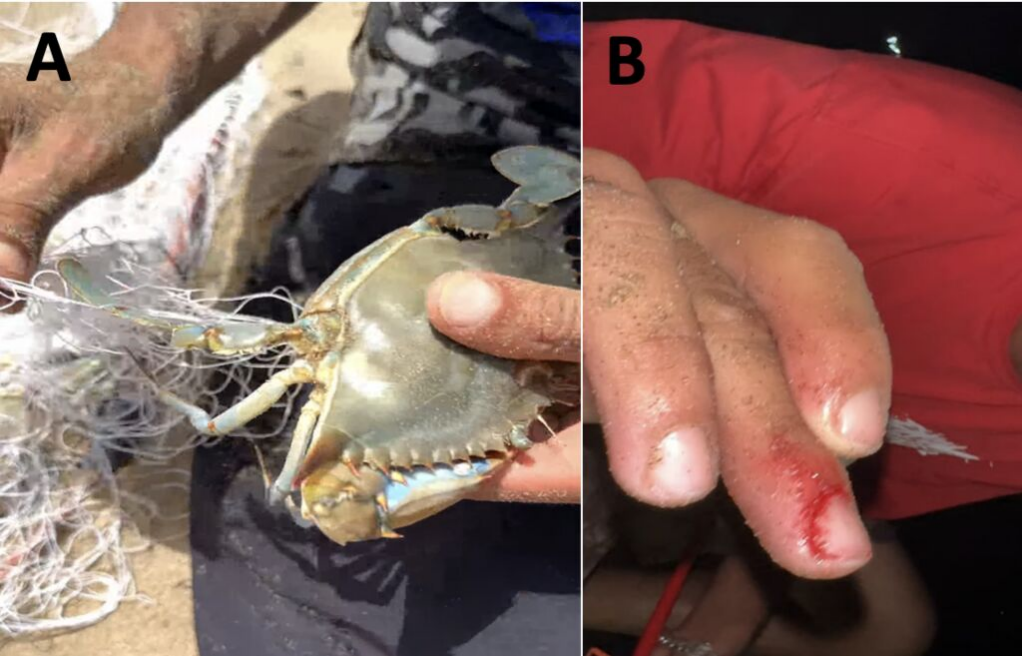
Damage caused by the *Callinectessapidus*. **A** on fishing nets; **B** injuries on fisherman when removing crabs from nets.

A total of 127 blue crabs (122 adult and 5 juveniles of indeterminate sex) were recorded between November 2022 and August 2023; 39 females (of which 23 were ovigerous) and 83 males were caught in brackish waters and off the coast at 1–7 m depth. The caught specimens' measurements were as follows for all locations and dates: Carapace length varying from 21.0 to 87.4 mm (mean 67 ± 13.3 mm), with a carapace width between 47.5 and 198.3 mm (mean 135.3 ± 38.7 mm), while the weight was ranging between 56.4 to 488.5 g (mean 193 ± 123.3 g) (Table 1). These were categorised using the system proposed by Harding (2003), which relies on carapace width for classifying blue crabs as small (CW < 80 mm), medium (CW 80–120 mm) and large (CW > 120 mm). Therefore, all sampled blue crab represent different stages of sexual maturity for both sexes, including immature and mature, ovigerous and non-ovigerous. Most of the specimen’s egg mass of ovigerous females was brownish and comprised smaller eggs (Fig. 2D). Males predominated, while females were normally a little smaller than males, but male sizes varied a little more than those of females. In addition, anecdotal comments were gathered from local experienced fishermen. They reported that, when the blue crabs were taken out of the trammel net, the crab spines and claws hurt them (Fig. 3B). Additionally, they expressed concerns regarding crab predation affecting their catches, which included a variety of high-value species, in addition to damage to their fishing gear (Fig. 3A). A reduction in productivity and fisheries value results from the need to discard catches damaged by the blue crab. This species, according to the fishermen, poses a risk to their fishing operations.

## Discussion

**Figure 4.**
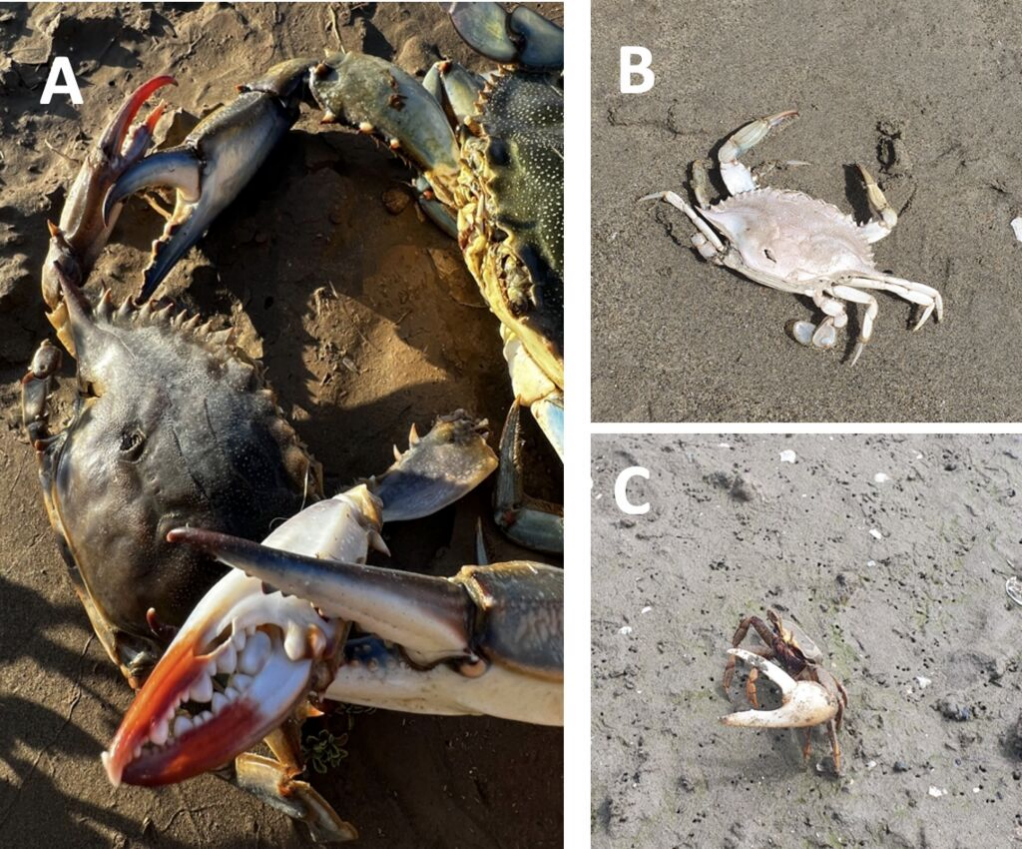
**A** Aggressive blue crab male in competition with a female one and shows cannibalism and scavenger behaviour; **B** Occurrence of molts in Oued Tani, Martil; **C** Native crab *Ucatangeri* in Oued Tani.

Blue crab populations differ significantly in terms of maximum carapace length (CL) and carapace width (CW) (Giraldes et al. 2016). As previously demonstrated in literature (Hajjej et al. 2016), there is substantial evidence that abiotic factors in connection with the climate of the region have the greatest effect on these parameters. The fact that multiple length and width classes are present year-round further demonstrates the species' established status in Oued Tani and Oued Negro. This is consistent with reports of the species' capacity for rapid establishment and spread in several parts of the world (Fuentes et al. 2019, Shaiek et al. 2021). The blue crab has an elevated reproductive capacity in which females lay 2- 8 million eggs produced during a single spawning event (Castriota et al. 2012), a great swimming ability (Diez and Jover 2015), rapid growth and short reproductive cycle (Mancinelli et al. 2013). The species is a successful invader because of these characteristics. The life cycle of the American blue crab is distinguished by the inclusion of marine and inshore brackish environments. The top and middle regions of estuarine systems are home to adult males who prefer brackish waters, whereas adult females who are ovigerous concentrate and spawn in more salty waters (Hines 2007). In order to guarantee safe and effective spawning in the absence of hostile male competition, female crab species migrate in their reproductive phase to deeper waters (Le Vay and Falamarzi 2009). This is an essential strategic adaption of these species because the adult male is omnivorous, a generalist predator and scavenger who occasionally engages in cannibalism (Gennaio et al. 2006, Diez and Jover 2015). These caracters are similar to what we found in ‘Oued tani’ Martil (Fig. 4A). New alien invasive species could have a negative impact on ecosystems and economically valuable activities like small-scale fisheries (Occhipinti-Ambrogi and Savini 2003). Many of these effects have measurable economic costs (Zenni et al. 2021). Blue crabs being trapped and entangled in fishing nets might lead to different losses such as mutilating fish in nets and damage to fishing gear (tearing trammel nets (Fig. 3A)) (Mancinelli et al. 2017b, Garcia et al. 2018, Kara and Chaoui 2021). In addition to the direct economic impacts, this species has the potential to have significant ecological effects, either through competitive interactions or predatory activities, with a variety of native fish and biota species (Labrune et al. 2019). The co-existence of the *C.sapidus* and the native crab *Ucatangeri* in the Oued Tani suggests that the two species might engage in competition in both space and trophic level within this aquatic ecosystem. The source of the *C.sapidus* individual discovered in the Oued Tani and Oued negro is uncertain, but since *C.sapidus* has been found in the Mediterranean Region, multiple hypotheses concerning the introduction mechanisms have been proposed. First, it is possible that the presence of the *C.sapidus* individuals found in Negro River (Fnideq) and Oued Tani (Martil) is due to ballast tanks (Klaoudatos and Kapiris 2014). In this sense, individuals belonging to this species have also been observed lately in the most important ports of western Morocco, the port of Tangier Med, which is located close to Fnideq (32 km of distance) and Martil (about 60 km). This suggests that the blue crab may be establishing enduring populations in this region. In addition, if we accept the hypothesis that the blue crab naturally can migrate by travelling up to several hundred kilometres (Morais et al. 2019), the population's most likely source of the study areas would presumably be Segura in Spain, south-western Mediterranean Sea (about 600 km), where several reports of the species have been made. However, the blue crab is quickly increasing its distribution on a global scale (Mancinelli et al. 2021). With readily available trophic resources (molluscs, crustaceans, annelids and commercially important fish), the proper ranges of salinity and temperature, as well as an appropriate environment to shelter its larvae, the river mouths of "Oued Tani" and "Oued Negro" offer the ideal conditions for the establishment and propagation of these invasive species. Once the invasive species has established, it becomes very challenging to stop the expansion. However, there are no established monitoring procedures in Morocco for blue crabs. Invasiveness of the Atlantic blue crab may be controlled by coastal communities by boosting captures for human consumption (Mancinelli et al. 2017a). In eastern Mediterranean countries, it is starting to gain commercial importance like in Egypt (Fatma et al. 2016) and Turkey (Ayas and Ozogul 2011). Regionally, Franke (2007) and Leone (2020) both released printed and online cookbooks recipes that promote the utilisation of non-indigenous species (NIS) as ingredients in meals. To further advertise this innovative product and promote its use, a number of sampling campaigns and culinary demonstrations have been held in Tunisia at different national and international events (Khamassi et al. 2022). These actions reduced blue crab damage and gave rise to new business dynamics and jobs.

## Conclusion

According to the current findings of this study, *Callinectessapidus* is well established in Morocco and has turned into a significant bycatch issue since it damages fishing gear. Nevertheless, in defiance of all the unfavourable effects, the blue crab might be advantageous in the recently invaded environments by maintaining a significant fishery along Morocco's coast if it is exploited and made available for commercialisation in Moroccan marketplaces. Therefore, to develop a management strategy for this invasive crab, comprising determining its inherent capacity as a new marine resource, further research on the biological and ecological characteristics of the *C.sapidus* population and its dispersion ability is thus necessary.

## Supplementary Material

7B06AEC3-F0FF-5895-AD49-558309951826 10.3897/BDJ.12.e115875.suppl1 Supplementary material 1 Data on the findings of Callinectessapidus Data type Table File: oo_934996.xlsx https://binary.pensoft.net/file/934996 Fatima Zahra Hamiche, Mustapha Aksissou

## Figures and Tables

**Table 1.** Morphometric measurements of males, females and juveniles of *Callinectessapidus* specimens from the western Mediterranean coast of Morocco. **N** number of captured specimens; **CL** Carapace length; **CW** Carapace width; **W** Body weight; **SD** standard deviation.

Sex	N	CW (mm)			CL (mm)			W (g)		
		Min	Max	Mean ± SD	Min	Max	Mean ± SD	Min	Max	Mean ± SD
Males Females Juveniles Total	83 39 5 127	91.4 80.6 47.5 47.5	198.3 182.2 71.3 198.3	145.4 ± 34.6 123 ± 35.3 61.7 ± 11.9 135.3 ± 38.7	53.7 48.1 21.0 21.0	87.4 85.7 38.2 87.4	70.7 ± 9.9 64.1 ± 12.2 29.7 ± 6.4 67 ± 13.3	71.4 57.3 56.4 56.4	488.5 264.1 80.5 488.5	238.5 ± 124.1 112.5 ± 62.8 66.6 ± 11.4 193 ± 123.3
